# β-Thujone and Its Derivatives Modify the Probing Behavior of the Peach Potato Aphid

**DOI:** 10.3390/molecules24101847

**Published:** 2019-05-14

**Authors:** Anna Wróblewska-Kurdyk, Radosław Gniłka, Katarzyna Dancewicz, Aleksandra Grudniewska, Czesław Wawrzeńczyk, Beata Gabryś

**Affiliations:** 1 Department of Botany and Ecology, University of Zielona Góra, Szafrana 1, 65-516 Zielona Góra, Poland; k.dancewicz@wnb.uz.zgora.pl (K.D.); b.gabrys@wnb.uz.zgora.pl (B.G.); 2 Department of Chemistry, Wrocław University of Environmental and Life Sciences, Norwida 25, 50-375 Wrocław, Poland; radoslaw.gnilka@port.org.pl (R.G.); aleksandra.grudniewska@upwr.edu.pl (A.G.); czeslaw.wawrzenczyk@upwr.edu.pl (C.W.); 3 Łukasiewicz Research Network–PORT Polish Center for Technology Development, Stabłowicka 147, 54-066 Wrocław, Poland

**Keywords:** α-thujone, β-thujone, Beckmann rearrangement, *Myzus persicae*, antifeedants

## Abstract

Thujone is a natural biologically active monoterpene ketone component of essential oils of numerous plants. The aim of the study was to evaluate the effect of β-thujone and β-thujone derivatives bisulfite adduct, lactone, oxime, and lactam application on behavior of *Myzus persicae* (Sulz.) (Hemiptera: Aphididae) during probing and settling. The choice and no-choice tests (aphid settling and Electrical Penetration Graph (EPG), respectively) revealed that stereochemistry of thujone was important for biological activity (β-thujone caused changes in aphid behavior while α-thujone did not) and that cyclopentane ring modifications and functional groups addition gave derivatives that possessed stronger and more durable deterrent effects. The most effective modification was the incorporation of a lactam moiety into the β-thujone molecule. Application of β-thujone lactam limited aphid settling for at least 24 h, caused restlessness in aphids and a delay or failure in reaching phloem phase by *M. persicae*. β-Thujone lactam can be considered a deterrent of medium potency with activity expressed at preingestive phase of aphid probing. Other compounds did not restrain aphid stylet penetration in non-phloem tissues but slightly limited sap ingestion (lactone, oxime), and restrained aphid settling for a period of less than 24 h (β-Thujone, bisulphite adduct, lactone).

## 1. Introduction

**Figure 1:**
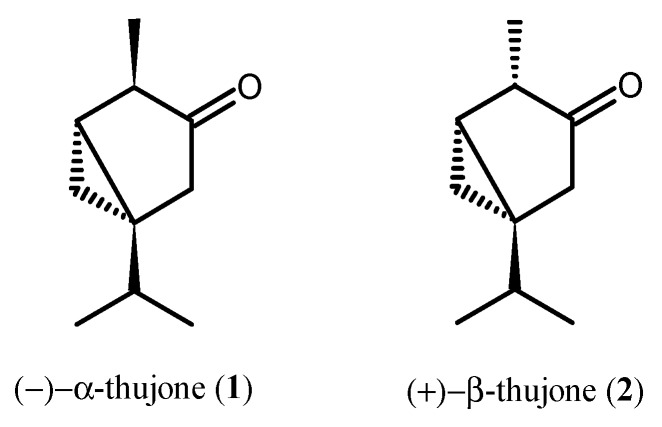
Diastereoisomers of thujone.

Thujone is a bicyclic monoterpene ketone that occurs naturally in two diastereoisomeric forms, (−)-α-thujone (**1**) and (+)-β-thujone (**2**), that differ in the stereochemistry of their configurations at C-4 (Figure 1) [1,2].

α-Thujone (**1**) and β-thujone (**2**) are major constituents and biologically active components of essential oils derived from a variety of plants of Asteraceae (e.g., tansy *Tanacetum vulgare* L., yarrow *Achillea millefolium* L., and various species of wormwood *Artemisia* L.), Cupressaceae (e.g., white cedar leaf *Thuja occidentalis* L. and common juniper *Juniperus communis* L.), Lamiaceae (e.g., sage *Salvia officinalis* L. and hyssop *Hyssopus officinalis* L.) [2,3,4,5]. The α to β ratio in plants is variable and depends on the plant source [6]. Thujone-containing plants have long been used in traditional medicine to treat various ailments, such as bronchial catarrh, enuresis, cystitis, psoriasis, uterine carcinomas, amenorrhea, and rheumatism [7]. Presently, there are numerous studies that point at the therapeutic use of both thujone isomers, for example, in treatment of polycystic ovary syndrome, cancer, and as a bioantimutagen [8,9,10]. Thujone has also been found to be a potent repellent, antifeedant, and insecticide to mammal and insect herbivores and mosquitoes [11,12,13,14,15,16,17]. 

The peach potato aphid *Myzus persicae* (Sulz.) (Hemiptera: Aphididae) is one of the most noxious insect pest species. It can infest plants of over 40 different families including many economically important ones worldwide, and it is able to transmit over 100 plant viruses [18]. At present, aphid control depends mainly on the use of neurotoxic insecticides. Due to the repeating applications, many aphid species, especially *M. persicae*, have developed resistance to several aphicides [19]. Therefore, an alternative method of aphid control is needed. One of the possible approaches is the use of plant extracts and plant-derived chemicals which would repel aphids or deter their feeding, and in consequence, reduce their number on plants. The use of natural compounds on a wide scale is costly. For this reason, there is a strong demand for synthetic, behavior modifying chemicals which are simple to obtain in the laboratory. Mono- and sesqui-terpenoids are promising groups of compounds that show significant deterrent effects on aphid probing and feeding [20,21]. Interestingly, there is a difference in aphid antifeedant activity between the isomers of a given compound: α-ionone was more active than β-ionone, *R*-pulegone was more active than *S*-pulegone, and *S*-limonene was more active than *R*-limonene [22]. *S*-Limonene inhibited phloem sap ingestion and reduced the number of phloem sap ingestion phases during *M. persicae* stylet penetration of plant tissues while *R*-pulegone did not and the chiral center configuration of the *S*-limonene-derived lactones was important in expression of feeding deterrent activity [23]. The biological activity of aphid antifeedants depends not only on the stereochemistry of the molecule but is related also to the presence of various functional groups. Simple modifications of natural terpenoid structures may enhance their activity. The introduction of the hydroxy group into the molecule of jasmonates led to biologically active derivatives that regulate the behavior of *M. persicae* by termination of their feeding [24]. Epoxy-ε-lactones that derived from (+)-dihydrocarvone were strong deterrents [25]. While β-damascone appeared a behaviorally inactive compound, the dihydro-β-damascol, β-damascone acetate, and β-damascone-derived δ-bromo-γ-lactone, and unsaturated γ-lactone were potent antifeedants that affected pre-phloem and phloem aphid probing activities [26]. The hydroxyderivatives, epoxyderivatives, and alkyl-substituted δ-lactones derived from *cis*-jasmone and dihydrojasmone hindered the foraging activity of *M. persicae* during early stages of probing at the level of non-phloem tissues [27].

Although the repellent, antifeedant, and insecticidal activity of thujone has been examined towards a number of herbivorous insects, the aphid response to this terpenoid has never received full attention. Nottingham and Hardie [28] have only found that *Aphis fabae* Scopoli (Hemiptera: Aphididae) was repelled by tansy odor. Symptoms of a likely deterrent effect of thujone on *M. persicae* behavior were observed by Dancewicz et al. [29] when the thujone-containing extracts of *Artemisia absinthium* and *T. vulgare* were applied to the leaves of Chinese cabbage *Brassica pekinensis* (Brassicaceae). According to the aphid settling assay results, *M. persicae* avoided the treated leaves during the period of at least 24 h past application [29]. 

In prologue to the present studies, we discovered that neither *T. occidentalis* essential oil (containing 69.80% of α-thujone and 9.47% of β-thujone) nor pure α-thujone (**1**), showed any activity towards the extremely polyphagous and insecticide-resistant *M. persicae* in aphid settling bioassay in contrast to pure β-thujone (**2**) that was a strong deterrent [Anna Wróblewska-Kurdyk and Beata Gabryś; aphid settling assay data presented in Section 2.2.1.] Therefore, we focused mainly on the behavioral background of the feeding deterrent activity of β-thujone and its derivatives by investigating aphid stylet penetration in plant tissues. The probing through plant non-vascular and vascular compartments with stylets of the piercing-sucking mouthparts is the crucial phase in the host-plant selection process of aphids. It allows the assessment of plant chemistry, which is the basic information about plant suitability for these herbivores [21]. The examination of aphid probing behavior by using the Electrical Penetration Graph (EPG) technique makes possible the location of the deterrent activity of natural as well as artificially applied chemicals within particular plant tissues and the association of this activity to particular phases of aphid probing [20,21,22,23,24,25,26,27]. Based on this information coupled with the results of the aphid settling assay, the potency and efficacy of prospective antifeedants may be assessed [20,21,22,23,24,25,26,27]. 

The main goal of the present study was to explore in detail the effects of β-thujone and β-thujone derivatives application on *M. persicae* behavior, which may indicate the possible uses of these compounds in the sustainable control of this aphid species. We were especially interested in which phases of aphid probing in plant tissues were, if at all, the most strongly affected by the application of β-thujone and its derivatives and what consequences this might have had on aphid settling. At the same time, considering the significance of alterations of natural terpenoid structures in the expression of antifeedant activity, the effects of cyclopentane ring modifications and functional groups addition to β-thujone molecule on behavioral responses of *M. persicae* during probing and settling have been investigated. 

## 2. Results

### 2.1. Synthesis of β-Thujone Derivatives

**Scheme 1:**
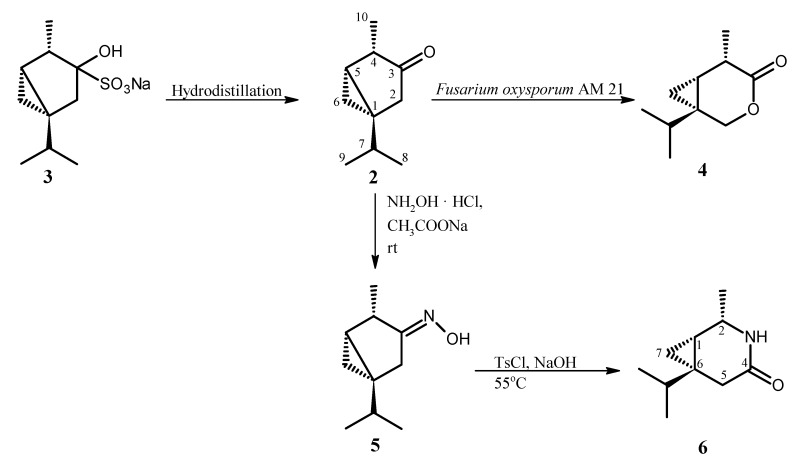
Synthesis of β-thujone lactone (**4**), oxime (**5**), and lactam (**6**).

The synthesis and structures of compounds studied in aphid settling (choice-test) and aphid probing behavior (no-choice test) experiments are presented in the Scheme 1.

β-Thujone (**2**) and lactone (**4**) were obtained as described previously [30,31]. β-Thujone (**2**) was isolated from essential oil of *Thuja occidentalis* L. as crystalline bisulfite adduct (**3**) which was hydrolyzed to β-thujone (**2**) by hydrodistillation with application of Deryng apparatus. β-Thujone lactone (**4**) was the product of microbial transformation of ketone (**2**) in the culture of *Fusarium oxysporum* AM21 (Scheme 1). The lactam (**6**) was obtained by Beckmann rearrangement of thujone oxime (**5**). Although oxime (**5**) and lactam (**6**) are known compounds [32], we would like to present new spectral (^1^H and ^13^C-NMR) data which were not published in the cited work [32].

In the first step, the reaction of β-thujone (**2**) with hydroxylamine hydrochloride afforded the crystalline oxime (**5**). The structure of oxime (**5**) obtained was established on the basis of its NMR spectral data. The signal of the C-3 carbon atom in the ^13^C NMR spectra of oxime (**5**) was found at 166.79 ppm and it was located at higher field in comparison to the signal of the same carbon atom (218.20 ppm) in the spectrum of starting ketone (**2**) [33]. Moreover, *δ* = 166.79 is characteristic for carbon atom which is connected with nitrogen atom. The *E* configuration of the imine bond was indicated by the chemical shift of the CH_2_-2 protons (2.34 and 2.79 ppm). These values are results of deshielding effect of *cis* oriented hydroxyl group. The presence of signals from protons CH_2_-6 at –0.05 ppm and 0.41 ppm in the ^1^H NMR spectrum of oxime (**5**) indicated that the cyclopropane ring was not affected during oximation of ketone (**2**). 

In the next step, oxime obtained (**5**) was subjected to the Beckmann rearrangement under conditions as described previously for synthesis of sabina lactams [34]. The structure of lactam obtained (**6**) was established also on the basis of its NMR spectral data. The one-proton signal at 3.85 ppm in ^1^H-NMR spectrum of lactam (**6**) was assigned to the H-2 proton. The value of its chemical shift indicated that this proton is connected to carbon atom directly bonded with nitrogen atom. This fact proved that the C-4 alkyl group in oxime (**5**) migrated to the nitrogen atom. The configurations 1*S*, 2*S*, 6*S* were assigned to chiral carbon atoms in lactam (**6**) taking into account the mechanism of Beckmann rearrangement as well as configuration of chiral center in β-thujone (**2**). The values of the chemical shifts of two one-proton multiplets at 0.31 and 0.52 ppm of CH_2_-7 indicated that the cyclopropane ring was not affected during the Beckmann rearrangement reaction.

### 2.2. Aphid Behavioral Studies

#### 2.2.1. Choice Test (Aphid Settling)

The application of β-thujone (**2**) and its derivatives: β-thujone lactone (**4**), β-thujone lactam (**6**), and bisulfite adduct (**3**) limited aphid settling at various degrees. The strongest deterrent effect occurred after application of lactam (**6**) and the deterrent effect of this compound was observed for at least 24 h after exposure, when the experiment was terminated. The deterrent effects of the remaining active compounds β-thujone (**2**), bisulfite adduct (**3**), and lactone (**4**) ceased two hours after exposure. *T. occidentalis* essential oil, α-thujone (**1**), and β-thujone oxime (**5**) did not affect the settling activity of *M. persicae* (Table 1).

#### 2.2.2. No-Choice Test (Aphid Behavior during Probing in Plant Tissues)

(1) General Aspects of Aphid Probing Behavior

**Figure 2:**
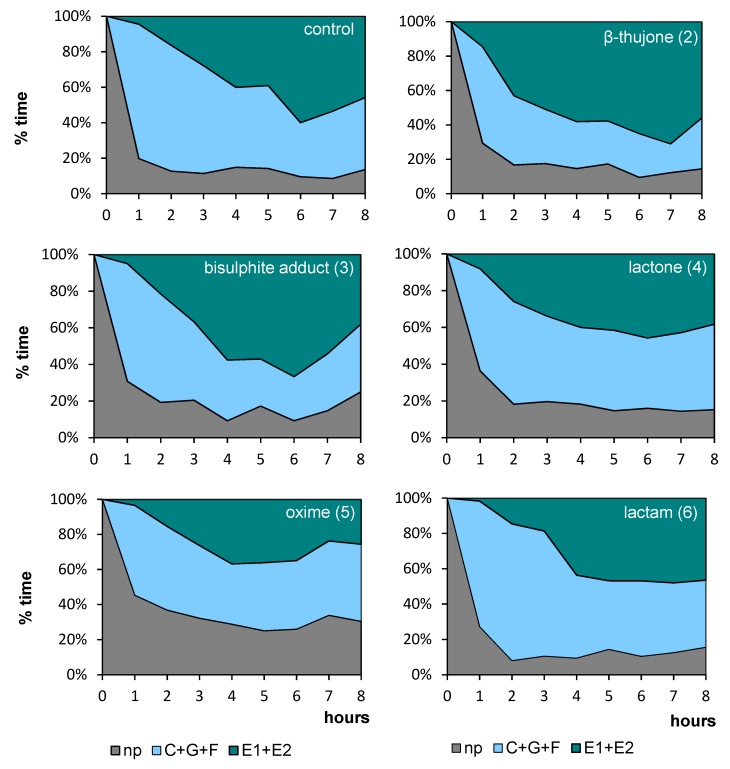
Temporal evolution of the probing behavior of *Myzus persicae* on *Brassica rapa* subsp. *pekinensis* treated with (+)-β-thujone (**2**) and its derivatives bisulfite adduct (**3**), lactone (**4**), oxime (**5**), lactam (**6**), expressed as the proportion of time spent on different stylet activities during the 8 hr Electrical Penetration Graph (EPG) experiment: np, non-probing; C+G+F, probing in non-phloem tissues (probing in non-vascular tissues + xylem phase + undefined stylet activities), E1+E2, total phloem phase (watery salivation + sap ingestion); The same plant was used to maintain the aphid stock culture; *n* = 15.

Regardless of the compound that was applied to the aphid host plant, all aphids started probing immediately after being given access to the plants at the beginning of the experiment. In the course of time, the trends in the proportion of time dedicated to individual phases of probing varied among aphids, depending on the compound applied. Generally, non-probing and pathway activities predominated during the initial 1–2 h of the experiment in all aphids on all plants (Figure 2). Later, on control, β-thujone (**2**), and bisulphite adduct (**3**)-treated plants, the proportion of non-probing phase decreased while the proportion of non-phloem and phloem probing phases in aphid activities increased, and at the end of the 8-h experiment, phloem phase was the main aphid activity on these plants, ranging from 46% on control to 56% on β-thujone (**2**)-treated plants. The proportion of phloem phase remained relatively low throughout the 8-h experiment on lactone (**4**), oxime (**5**), and lactam (**6**)-treated plants. The proportion of non-probing activities was relatively high on oxime (**5**)-treated plants throughout the 8-h experiment, as compared to other treatments (Figure 2).

The application of oxime (**5**) caused a decrease in the total duration of probing, an increase in the mean duration of individual probes, and a decrease in the number of sustained phloem sap ingestion phases in relation to control (**C**). The application of lactam (**6**) caused an increase in the total number of probes in relation to β-thujone (**2**)-treated plants, a decrease in sustained phloem sap ingestion phases in relation to control (**C**) plants, and a decrease in the number of probes containing the phloem phase and in the number of phloem phases in relation to control (**C**) and β-thujone (**2**)-treated plants. The application of bisulphite adduct (**3**) caused a slight increase in the total number of probes in relation to control (**C**). On β-thujone (**2**) and lactone (**4**)-treated plants, no significant differences in the values of parameters describing general aspects of aphid probing behavior in relation to control occurred (Table 2).

(2) Aphid Probing Behavior Prior to the First Phloem Phase

The time to reach the first phloem phase was 2.3 and 2.5 h longer on lactam (**6**)-treated than on control (**C**) and β-thujone-treated plants, respectively (Table 2). On lactam (**6**)-treated plants, the majority of all probes occurred before the first phloem phase and they were usually shorter than 3 min (Table 2). Aphid behavior on β-thujone (**2**) and its derivatives bisulfite adduct (**3**), lactone (**4**), oxime (**5**)-treated plants did not differ significantly from control during the period preceding the first occurrence of phloem phase (Table 2). 

(3) Aphid Probing Behavior during Phloem Phase

The proportion of aphids that reached phloem phase during the 8-h experiment and the proportion of phloem phase during probing were the lowest on lactam (**6**)-treated plants (60%) while in the remaining treatments this value ranged from 73% (oxime (**5**) treatment) to 100% (lactone (**4**) treatment) (Table 2). However, in aphids that did show phloem phase on lactam (**6**)-treated plants, the duration of the first phloem phase and the mean duration of an individual period of sap ingestion were the longest, six times longer than on control (**C**) plants, and the proportion of salivation during the phloem phase was the lowest (1%) (Table 2).

(4) ‘Exploratory Cell Punctures’ in Non-Phloem Tissues

The proportion of probes that included exploratory ‘short’ and ‘long’ cell punctures (pd-S and pd-L, respectively) was by 20–39% lower on oxime (**5**) and lactam (**6**)-treated plants than on control. The total number of pd-S exploratory cell punctures was 1.5 times lower and the mean number of pd-S per probe was lower on lactam (**6**) treated plants. The cumulative durations of subphases II-1 and II-3 in all pd-S were 2.3 and 1.8 times shorter on lactam (**6**) treated plants in relation to control. The mean duration of an individual pd-S was lower on β-thujone (**2**)**,** bisulphite adduct (**3**)**,** and lactone (**4**)-treated plants. In pd-L exploratory cell punctures, the only significant difference in relation to control was in the duration of subphase II-3, which was twice as short on lactam (**6**) treated plants as in aphids on control plants. On lactam (**6**)-treated plants, the proportion of probes with potential drops and the number of pd-S during one penetration were lower in relation to β-thujone (**2**)-treated plants (Table 3). 

(5) Structure–Activity Aspects

Within the pair of stereoisomers studied, α-thujone (**1**) was not active while β-thujone (**2**) was a weak deterrent for *M. persicae*. β-Thujone (**2**) showed deterrent properties for 2 h with no effect on aphid probing while the lactam derivative (**6**) was active for at least 24 h and affected probing in non-phloem tissues (Table 1, Table 2 and Table 3).

## 3. Discussion

In the present study, the deterrent activity of the studied compounds was examined in two independent complementary experiments, one involving freely moving aphids (aphid settling in a choice test) and the other involving tethered aphids (aphid probing behavior in the EPG no-choice test). The aim of the choice test was to show aphid preferences for the food source and the aim of the no-choice test was to explain the background of the observed preferences. 

The choice test, which lasted for 24 h, showed that the potency and durability of deterrent effect on *M. persicae* settling activity after exposure to *T. occidentalis* essential oil (α-and β-thujone, 69.80% and 9.47% respectively), α-thujone (**1**), β-thujone (**2**), and β-thujone derivatives bisulfite adduct (**3**), lactone (**4**), oxime (**5**), lactam (**6**) depended on the compound applied to the leaves of the host plant. Firstly, the application of *T. occidentalis* essential oil and its major component α-thujone (**1**) did not affect aphid settling in contrast to the other component of the essential oil, β-thujone (**2**), the application of which caused the avoidance of leaves by *M. persicae*. The seven-fold higher content of the non-deterrent α-thujone (**1**) as compared to the content of the deterrent β-thujone (**2**) in the essential oil studied was probably the reason why the freely moving aphids did not respond to the application of this oil. It is likely that the amount of β-thujone (**2**) was too low in the essential oil to evoke changes in *M. persicae* settling behavior. Secondly, on plants treated with β-thujone (**2**), bisulphite adduct (**3**), and lactone (**4**), aphids ceased to respond to these compounds two hours after application. The decrease in, and/or the loss of the deterrent activity of compounds administered to aphids via their host plants were observed also in our previous studies, after application of other terpenoids, e.g., in the case of piperitone and its derivatives and *cis*-jasmone and dihydrojasmone and their derivatives [27,35]. It is likely that similar reasons might have caused the observed phenomenon in the present study: the degradation of the compounds by plant enzymes, the translocation to other plant parts via phloem vessels, or other physiological/metabolic processes in the plant or in the aphid, all of which might make the concentration of the compound low and undetectable by the insect [35,36]. 

The electronic registration of aphid probing behavior (EPG technique) was applied in the present study as a no-choice test. The 8 h of continuous monitoring allowed the analysis of aphid behavior during pre-ingestive (within non-phloem tissues before the first phloem phase) phase of probing separately from the ingestive (within the phloem) phase of probing [27]. The disturbance of aphid probing at non-vascular level is usually manifested in frequent withdrawal of stylets from plant tissues, following probes of short duration. A high proportion of probes shorter than three minutes demonstrates notable restlessness of aphids before they could reach phloem vessels and that these probes do not reach beyond epidermis [37]. Such effect was observed in *M. persicae* on lactam (**6**)-treated plants. The limitation of aphid activities within the phloem tissue, that is a lower proportion of phloem phase during probing, less numerous sap ingestion bouts and a shortened duration of an individual sap ingestion phases indicate the deterrent properties of the applied substances which penetrated plant tissues as far as the vascular bundle and into the phloem sap. Such changes in the feeding behavior in contrast to the suitable food source are typical in aphids confronted with plant resistance [38,39,40,41]. In the present study, similar effects occurred in *M. persicae* on lactone (**4**), oxime (**5**), and lactam (**6**)-treated plants. 

Probing in non-phloem tissues is an obligatory stage during aphid mouthpart’s stylets’ plant penetration and an essential phase required for finding sieve elements in the phloem tissue that contain plant sap, the basic food for aphids. The exploration of non-phloem tissues that separate the aphid from the phloem includes brief cell punctures, called ‘exploratory cell punctures’ here, that serve as an opportunity to ingest small samples of cytoplasm for gustatory purposes in host plant recognition process [42]. The cell punctures are visualized as potential drops in EPG recordings. The so-called ‘long’ potential drops (L-pd) have been correlated with the transmission of viral diseases in potato by aphids [43]. The frequency of ‘short’ and ‘long’ cell punctures (potential drops) and the duration of ‘long’ cell punctures were associated with effectiveness of transmission of *Cucumber mosaic virus* (CMV) by *Aphis gossypii* Gloger [44]. The greater the number of cell punctures in non-phloem tissues and the longer were the II-1 and II-3 subphases, the more effective was the transmission of *Zucchini yellow mosaic virus* (ZYMV) by *M. persicae* [45]. The present study showed that the application of lactam (**6**) may potentially reduce the effectivity of non-persistent virus transmission. However, a study dedicated to virus transmission will be needed to precisely explore this possibility.

Structure–activity relationships were one of the aims of the present study. The analysis of aphid behavior at different time intervals after application allowed the selection of the strongest biologically active compounds and the characterization of the deterrent effect in terms of durability. Firstly, it has been demonstrated that stereochemistry of the natural starting compound was important for biological activity (β-thujone (**2**) modified aphid behavior while α-thujone (**1**) did not). Secondly, the structural changes in the molecule of β-thujone (**2**) applied in the present study gave the derivatives that possessed stronger and more durable deterrent effects, although the plant/insect metabolic aspects of the wide-ranging aphid responses to these compounds remain unknown, as discussed earlier. The most effective modification in the present study was the incorporation of lactam moiety into β-thujone (**2**) molecule. In comparison to aphids on β-thujone (**2**)-treated plants, tethered aphids on lactam (**6**)-treated plants reached phloem vessels much later or failed to do so within eight hours and the free aphids consequently avoided the treated plants during a period of at least 24 h.

When a practical application of a chemical is considered, especially for use for protection of food, the toxicity has to be taken into account. Thujone is believed potentially toxic to humans and the presence of α- (**1**) or β-thujone (**2**) in food and beverages is regulated by law in several countries [46,47] due to its neurotoxic activity: both α- (**1**) and β-thujone (**2**) act as noncompetitive blockers of the γ-aminobutyric acid (GABA)-gated chloride channel [2,5]. It is not yet known if both isomers exhibit the same biological effects [3]. However, α-thujone (**1**) has been reported about two to three-fold more active than β-thujone (**2**) [48] because it is more potent at the GABA-gated chloride channel [49,50]. β-Thujone (**2**), as the principal bioactive ingredient of wormwood oil, has been regarded a neurotoxic principle in absinthe but recent studies indicate that the toxic symptoms after consumption of absinthe were mainly due to its alcohol content [6]. Recent findings also show that the neuronal effects of thujone are completely reversible [48]. In mammals, thujone is quickly metabolized by liver microsomes [46]. Moreover, the observed effects of monoterpenes in bacteria and mammalian cells are consistent with hormesis phenomenon, characterized by a low dose beneficial effect and a high dose adverse effect of a stressor agent, provide a basis for further study of both chemopreventive and chemotherapeutic potential of camphor, eucalyptol and thujone [51].

Secondary plant metabolites have multiple functions in the herbivore–plant relationships, which makes them attractive targets for plant breeding. Molecular strategies leading to the modification or supplementation of the existing biosynthetic pathways of secondary metabolites in plants are under investigation [52]. Volatile oils easily penetrate membranes as non-polar (and therefore lipophilic) compounds. This quality is utilized in aromatherapy [1]. Our experiments demonstrated that exogenously applied β-thujone (**2**) and β-thujone derivatives (**3**–**6**) may penetrate the plant cuticle and epidermis, pass into deeper tissue layers, and in consequence, interfere in the instinctive process of plant selection by an aphid. Likewise, the exogenous application of camphene- and β-ionone to plants, made aphids reluctant to continue probing and feeding: the proportion of non-probing relative to other stylet activities was high, the success rate in reaching sieve elements and feeding was low, and the proportion of salivation in phloem phase was high [21]. We demonstrated also that the transcuticular application of β-thujone (**2**) and certain β-thujone-derived monoterpenoids bisulfite adduct (**3**), lactone (**4**), oxime (**5**), lactam (**6**) caused disturbances in plant recognition and acceptance, which may finally reduce aphid infestation. 

Considering the tissular localization of deterrent activity determined in the EPG experiments and the potency and durability of the deterrent effects determined in aphid settling test, the compounds studied can be divided into groups of varying activities. β-Thujone lactam (**6**) can be defined as a deterrent of medium potency, with activity expressed at preingestive phase of aphid probing. Although β-thujone lactam (**6**) limited aphid settling for at least 24 h, it disturbed aphid probing only in non-vascular tissues. However, the reluctance to probe beyond outer layers of mesophyll may contribute to the limitation of the transmission of semi-persistent and persistent viruses [27], which makes the β-thujone lactam (**6**) a promising compound in this context. β-Thujone (**2**), β-thujone bisulphite adduct (**3**), and β-thujone lactone (**4**), can be defined as weak deterrents which did not limit or slightly limited aphid stylet penetration in phloem tissues and did not limit or slightly limited aphid settling. α-Thujone (**1**) did not affect aphid behavior. 

## 4. Material and Methods

### 4.1. Synthesis of Compounds Studied

#### 4.1.1. General Methods

Reaction reagents: hydroxylamine hydrochloride and *p*-toluenesulfonyl chloride were purchased from Sigma-Aldrich (St. Louis, MO, USA). The starting material β-thujone (**2**) was obtained as described previously [30,31]. Analytical TLC was performed on aluminum plates coated with silica gel (Kiesielgel 60, F_254_, Fluka). All compounds were detected by spraying the plates with solution of 1% Ce(SO_4_)_2_, 2% H_3_[P(Mo_3_O_10_)_4_] in 10% aqueous H_2_SO_4_, besides lactam (**6**), which was detected by solution of ninhydrin 0.3% (w/v) in *n*-butanol. Silica gel (Kiesielgel 60, 230–400 mesh, Merck) column chromatography with a mixture of hexane, acetone and chloroform (3:1:1 v/v) as eluent was used to purification of lactam (**6**). Gas chromatography analyses were carried on an Agilent 7890A (GC System, Santa Clara, CA, USA) equipped with FID detector and H_2_ as carrier gas. Substrates and products of reaction mixtures were separated on capillary columns: HP-5 (30 m × 0.32 mm ID × 0.25 μm film) with followed oven temperature program 110 °C, 200 °C (10 °C min^−1^), 300 °C (30 °C min^−1^) (3 min) for lactam (**6**) and CP-ChiralSil-L-Val (25m × 0.25 mm ID × 0.12 μm film) with followed oven temperature program 80 °C, 120 °C (1.5 °C min^−1^) (2 min), 200 °C (15 °C min^−1^) (3 min) for oxime (**5**). ^1^H, ^13^C and two-dimensional (1H-1H COSY, 1H-13C HSQC) NMR spectra were recorded for CDCl_3_ on a Bruker Avance DRX 300 MHz. Chemicals shifts were referenced to the residual solvent signal (δ_H_ = 7.26, δ_C_ = 77.16). Melting points were determined on a Boetius apparatus.

#### 4.1.2. Synthesis of β-Thujone Oxime (**5**)

The β-thujone (**2**) (100 mg, 0.66 mmol) dissolved in methanol (5 mL) was added to 7 mL of aqueous solution of hydroxylamine hydrochloride (100 mg, 1.44 mmol) and sodium acetate (200 mg, 2.44 mmol). The reaction mixture was stirred for 48 h at room temperature, followed by the oxime (**5**) which was extracted with diethyl ether (3 × 25 mL). The combined organic phases were washed by aqueous saturated solution of sodium bicarbonate, dried on anhydrous MgSO_4_, and concentrated under vacuum. The pure crystal of β-thujone oxime (**5**) (100 mg, yield 91%) was obtained. The physical and spectral data of this product are given below:

*β-Thujone oxime* (**5**). Colorless crystals; m.p. 48–50 °C, lit. 53–54 °C [31]; ^1^H-NMR (300 MHz, CDCl_3_) δ: −0.05 (dd, *J* = 5.1 and 4.2 Hz,1H, one of the CH_2_-6), 0.41 (m, 1H, one of the CH_2_-6), 0.90 and 0.96 (two d, *J* = 6.9 Hz, 6H, (CH_3_)_2_CH-), 1,09 (d, *J* = 6.6 Hz, 3H, CH_3_-10), 1.25 (m, 1H, H-5), 1.42 (septet, *J* = 6.9 Hz, 1H, (CH_3_)_2_CH-), 2.34 (dd, *J* = 17.8 and 2.1 Hz,1H, one of the CH_2_-2), 2.79 (dd, *J* = 17.8 and 1.5 Hz,1H, one of the CH_2_-2), 2.99 (m, 1H, H-4), 8.93 (broad s, 1H, -OH); ^13^C-NMR (150 MHz, CDCl_3_) δ: 13.21 (C-6), 14.80 (CH_3_-10), 19.88 and 19.96 ((CH_3_)_2_CH-), 26.49 (C-5), 30.08 (C-1), 30.77 (C-2), 32.23 ((CH_3_)_2_CH-), 39.27 (C-4), 166.79 (C-3).

#### 4.1.3. Synthesis of β-Thujone Lactam (**6**)

The β-thujone oxime (**5**) (100 mg, 0.6 mmol) was dissolved in acetone (1 mL) and added dropwise to the stirred aqueous solution (2 mL) of sodium hydroxide (60 mg, 1.5 mmol) at room temperature. Next, the reaction mixture was heated (glycerin bath) under reflux to 55 °C and 1 mL of acetone solution of *p*-toluenesulfonyl chloride (160mg, 0.84 mmol) was added. After 4 h, the reaction mixture was cooled to room temperature and then acetone was evaporated in vacuo. The Beckmann rearrangement product (**6**) was extracted with diethyl ether (3 × 25 mL). The combined organic phases were washed with aqueous saturated solution of sodium bicarbonate, dried on anhydrous MgSO_4_, and concentrated under vacuum. The β-thujone lactam (**6**) was purified by silica-gel column chromatography (hexane-acetone-chloroform 3:1:1, v/v). Pure β-thujone lactam (**6**) (53.4 mg, yield 49%) was obtained. Its physical and spectral data are given below. 

*β-Thujone lactam* (**6**) (1*S*,2*S*,6*S*)-2-methyl-6-(propan-2-yl)-3-azabicyclo[4.1.0]heptan-4-one). Colorless crystals, m.p. 85–88 °C, lit. 89–90 °C [31]; ^1^H-NMR (300 MHz, CDCl_3_) δ: 0.31 (ddd, *J* = 8.4, 5.3 and 1.4 Hz, 1H, one of the CH_2_-7), 0.52 (m, 1H, one of the CH_2_-7), 0.91 and 0.94 (two d, *J* = 6.7 Hz,6H, (CH_3_)_2_CH-), 1.12 (m, 1H, (CH_3_)_2_CH-), 1.19 (d, *J* = 6.3 Hz,3H, CH_3_-2), 2.36 (dd, *J* = 16.8 and 1.1 Hz, 1H, one of the CH_2_-5), 2.46 (d, *J* = 16.8 Hz, 1H, one of the CH_2_-5), 3.85 (qd, *J* = 6.3 and 2.6 Hz, 1H, H-2), 6.08 (broad s, 1H, -NH); ^13^C-NMR (150 MHz, CDCl_3_) δ: 9.11 (C-7), 18.60 and 19.09 ((CH_3_)_2_CH-), 21.59 (CH_3_-2), 23.36 (C-1), 24.04 (C-6), 31.02 (C-5), 35.55 ((CH_3_)_2_CH-), 46.16 (C-2), 172.32 (C-4).

### 4.2. Aphids

#### 4.2.1. Aphid and Plant Cultures

The laboratory culture of *M. persicae* was maintained on Chinese cabbage *Brassica rapa* L. ssp. *pekinensis* L. *M. persicae* probing behavior was monitored on *B. rapa* ssp. *pekinensis* (the plant it was reared on), which was considered as a control plant. Aphids and plants were kept in the laboratory at 20 °C, 65% relative humidity, and L16:8D photoperiod. The stock colonies were kept as follows: an adult aphid was placed on a plant and allowed to reproduce for 24 h. Then the female was removed and the remaining larvae were allowed to develop. Young, 2–3 days old (i.e., 2–3 days after the final moult) viviparous apterous females were selected for experiments. The plants used in the bioassays were 5–6 weeks old.

#### 4.2.2. Choice-Test (Aphid Settling)

Aphids settle on a plant only when they accept it as a food source. Therefore, the number of aphids that settle and feed on a given substrate is a good indicator of host quality. A conventional settling choice test was conducted to evaluate the deterrent activities of studied extracts after Powell et al. [53]. This bioassay allows studying aphid host preferences under semi-natural conditions. Leaves cut from cabbage plants, were dipped for 10 s in the 0.1% solution of a studied compound dissolved in 70% ethanol or control solution (70% ethanol) and dried in the air for 1 h at room temperature. Two leaves (treated and control) were transferred to Petri dishes. Afterwards, 20 apterous females of *M. persicae* were placed between the leaves at the center of the Petri dish (the distances between two leaves approximately 2.0 cm). Aphids were offered a choice between treated and control leaves. Aphids that settled on each leaf were counted at 1, 2 and 24 h intervals after the beginning of the experiment. This experiment was replicated 8 times for each treatment (total number of insects per treatment = 160). The number of aphids that settled on treated leaves was compared to the number of aphids that settled on control leaves 1, 2, and 24 h after treatment, separately for each time point; data were analyzed using Student t-test (*p* ≤ 0.05).

#### 4.2.3. No-Choice Test (Electronic Registration of Aphid Probing Behavior)

Aphid probing behavior was monitored using the technique of electronic registration of aphid probing in plant tissues, known as EPG [54]. The analysis of parameters derived from EPG (frequency, duration and sequence of different waveforms) reflect behavioral responses of aphids to differences in plant acceptability [55,56,57,58]. The basic principle of this technique is that the aphid and plant are made parts of an electric circuit, which is completed when the aphid inserts its stylets into the plant. Weak voltage is supplied in the circuit, and all changing electric properties are recorded as EPG waveforms that can be correlated with aphid activities and stylet position in plant tissues [59]. In our study, an aphid was attached to a golden wire electrode (1.5–2.0 cm long, 0.18 μm diam.) with conductive water-based silver paint (EPG-Systems, Dillenburg 126,703 CJ Wageningen, The Netherlands) and starved for 1 h prior to the experiment. Probing behavior of 15 apterous females per studied plant/aphid combination was monitored for 8 h continuously with the four-channel and eight-channel DC EPG recording equipment. The incomplete (i.e., shorter than 8 h) recordings were excluded from analysis. Each aphid was given access to a freshly prepared plant: one leaf of a plant was covered with the studied compound or a solvent (control). The leaf was dipped for 10 s in the 0.1% solution of the studied compound dissolved in 70% ethanol or control solution (70% ethanol) and dried in the air for 1 h at room temperature. The plant electrode was placed in the soil. The length of the golden wire electrode and the position of EPG probe during the experiment (aphid + electrode + pre-amplifier) were adjusted to prevent the aphid contact with the untreated parts of the plant. Signals were saved and analyzed using the PROBE 3.1 software provided by W. F. Tjallingii (EPG-Systems, Dillenburg 126,703 CJ Wageningen, The Netherlands). The following aphid activities were distinguished: np—non probing, C—pathway (probing in non-vascular tissues), G—xylem phase, F—undefined stylet activities, E—phloem phase (divided into E1 and E2 that represent watery salivation and sap ingestion, respectively) [60]. Waveforms F and G occurred rarely, therefore were included with waveform C as non-phloem phase probing activities and analyzed as such in all calculations. Additionally, the characteristic potential drops (‘pd’) during waveform C were analyzed. These potential drops, called ‘exploratory cell punctures’ in the present work, represent short punctures of plasmalemma of cells [60]. The potential drops consist of three phases: phase I represents the puncture of cell by the stylets, phase II—activity within the cell, and phase III—withdrawal of stylets. Phase II is divided into three subphases: subphase II-1 represents egestion of saliva into the cell, which can be associated with inoculation of non-persistent viruses and subphase II-3 represents ingestion of cell contents, which may be associated with acquisition of the viruses. The meaning of the subphase II-2 is unknown [44,45,61,62]. Basing on the number of pulses within subphase II-3, the pds are divided into pd-S (‘short’ pd, with 0–2 pulses) and pd-L (‘long’ pd, with >3 pulses) [62]. In the present work, the frequency and duration of pd-S and pd-L were analyzed due to their importance in the transmission of plant viral diseases.

All EPG parameters describing aphid probing behavior were calculated manually and individually for every aphid using the EPG analysis Excel worksheet created for this study by one of the authors (Anna Wróblewska-Kurdyk). Subsequently, the mean and standard errors were determined. Aphid behavior on leaves treated with β-thujone was compared to aphid behavior on control plants. Aphid behavior on leaves treated with β-thujone derivatives was compared to aphid behavior on control plants and on β-thujone treated plants, separately. Mann–Whitney U-test was used for these comparisons. All statistical calculations were performed using StatSoft, Inc. (2014) STATISTICA (data analysis software system), version 12, www.statsoft.com.

## Figures and Tables

**Table 1:** Effect of *T. occidentalis* essential oil, (‒)-α-thujone (**1**), (+)-β-thujone (**2**) and β-thujone (**2**) derivatives bisulfite adduct (**3**), lactone (**4**), oxime (**5**), lactam (**6**), on settling of *Myzus persicae* on *Brassica rapa* subsp. *pekinensis*.

Compounds		Mean Number of Aphids		
		1 h	2 h	24 h
Essential oil (α-thujone + β-thujone)	test	4.9 (±1,3)	3.9 (±1.1)	5.8 (±1.6)
	control	8.3 (±1.1)	4.5 (±0.9)	4.1 (±0.8)
	p	0.0690	0.6795	0.3806
α-thujone (**1**)	test	8.3 (±0.6)	6.0 (±1.0)	2.8 (±0.6)
	control	6.1 (±0.9)	3.8 (±0.8)	3.0 (±2.8)
	p	0.0827	0.1036	0.7645
β-thujone (**2**)	test	3.8 (±0.5)	3.6 (±0.4)	3.1 (±0.5)
	control	10.8 (±0.9)	7.3 (±1.1)	3.0 (±0.5)
	p	0.0000	0.0081	0.8643
Bisulfite adduct of β-thujone (**3**)	test	2.3 (±0.4)	1.9 (±0.5)	1.2 (±0.5)
	control	5.0 (±0.7)	4.6 (±0.6)	1.9 (±0.7)
	p	0.0175	0.0029	0.4628
Lactone of β-thujone (**4**)	test	3.5 (±0.8)	1.5 (±0.6)	1.2 (±0.5)
	control	9.1 (±1.1)	5.1 (±0.7)	2.3 (±1.1)
	p	0.0012	0.0017	0.3578
Oxime of β-thujone (**5**)	test	5.1 (±1.1)	3.1 (±1.2)	1.5 (±0.3)
	control	7.5 (±1.1)	5.8 (±0.9)	2.9 (±0.6)
	p	0.1619	0.1074	0.0582
Lactam of β-thujone (**6**)	test	2.0 (±0.5)	1.5 (±0.5)	1.6 (±0.4)
	control	10.3 (±1.1)	8.0 (±0.7)	3.8 (±0.8)
	p	0.0000	0.0000	0.0300

Numbers represent the mean number of aphids that settled on the treated (test) and control leaves (±SD). Student t-test at *p* = 0.05 was used to compare the number of aphids on treated and control leaves at each time point, separately.

**Table 2:** Probing behavior (EPG parameters) of *Myzus persicae* on *Brassica rapa* subsp. *pekinensis* leaves treated with (+)-β-thujone (**2**) and its derivatives: bisulfite adduct (**3**), lactone (**4**), oxime (**5**), lactam (**6**) ^A^. The same plant was used to maintain the aphid stock culture.

EPG Parameter	Compounds					
	Control	2	3	4	5	6
**General Aspects of Aphid Probing Behavior**						
	*n* = 15	*n* = 15	*n* = 15	*n* = 15	*n* = 15	*n* = 15
Total duration of non-probing ^a^ (h)	1.0 (±1.3)	1.5 (±2.0)	1.4 (±1.3)	1.5 (±1.1)	2.6 * (±2.1)	1.1 (±1.1)
Total duration of probing in non-phloem tissues ^b^ (h)	4.1 (±2.1)	2.7 (±1.9)	3.2 (±1.4)	3.7 (±2.1)	3.5 (±1.9)	3.9 (±2.5)
Total duration of phloem phase ^c^ (h)	2.9 (±2.3)	3.8 (±2.6)	3.4 (±2.0)	2.8 (±2.6)	1.9 (±2.4)	3.0 (±3.0)
Total number of probes ^d^ (#)	24.7 (±9.8)	20.7 (±16.8)	27.1 * (±12.2)	35.9 (±23.2)	38.3 (±26.8)	39.9 ^+^ (±35.2)
Mean duration of a probe ^d^ (min)	21.7 (±17.2)	37.0 (±43.6)	30.3 (±58.1)	39.3 (±67.7)	40.6 * (±80.9)	16.9 (±12.1)
Duration of first probe ^d^ (min)	1.8 (±4.3)	3.6 (±7.0)	1.8 (±3.3)	3.2 (±6.5)	0.9 (±1.4)	11.1 (±41.3)
Number of probes with phloem phase ^d^ (#)	1.9 (±1.0)	1.9 (±1.4)	2.2 (±1.5)	2.5 (±1.5)	1.7 (±1.6)	0.7 *^,+^ (±0.7)
Proportion of aphids reaching phloem phase (#)	0.9 (±0.3)	0.9 (±0.3)	0.9 (±0.3)	1.0 (±0.0)	0.7 (±0.5)	0.6 (±0.5)
Time from first probe to first phloem phase (h)	2.4 (±2.1)	2.2 (2.0)	2.0 (±1.9)	2.1 (±2.0)	3.4 (±3.1)	4.7*^,+^ (±3.0)
Number of phloem phases (#)	4.7 (±3.0)	3.3 (±3.2)	5.6 (±4.8)	4.7 (±3.3)	3.5 *^,+^ (±3.8)	1.0 (±1.1)
Number of sustained sap ingestion phases ^e^ (#)	2.1 (±1.7)	1.4 (±1.2)	2.7 (±1.9)	1.6 * (±1.2)	0.9 * (±1.1)	0.9 (±1.1)
Phloem phase index ^f^ (#)	0.4 (±0.3)	0.5 (±0.3)	0.5 (±0.3)	0.4 (±0.3)	0.3 (±0.3)	0.4 (±0.4)
Phloem salivation index ^g^ (#)	0.1 (±0.1)	0.1 (±0.1)	0.04 (±0.1)	0.1 (±0.1)	0.1 (±0.2)	0.01 *^,+^ (±0.04)
**Activities in Non-Phloem Tissues before the First Phloem Phase**						
	*n* = 14	*n* = 15	*n* = 14	*n* = 15	*n* = 11	*n* = 9
Total duration of non-probing before first phloem phase ^B^ [h]	0.4 (±0.3)	0.6 (±1.1)	0.5 (±0.5)	0.8 (±1.1)	0.9 (±1.1)	0.7 (±0.8)
Total duration of probing in non-phloem tissues before first phloem phase ^B^ (h)	1.6 (±1.3)	1.3 (0.9)	1.2 (±0.7)	1.4 (±1.2)	1.1 (±0.8)	1.9 (±1.2)
Number of probes before first phloem phase (#)	13.2 (±9.6)	10.4 (±7.4)	9.4 (±5.3)	13.2 (±15.8)	11.2 (±10.7)	33.3 *^,+^ (±35.4)
Number of probes < 3 min. before first phloem phase (#)	8.0 (±6.5)	6.1 (±5.3)	5.7 (±4.5)	8.4 (±10.1)	9.5 (±10.1)	27.9 *^,+^ (±30.4)
**Activities in Sieve Elements**						
	*n* = 14	*n* = 15	*n* = 14	*n* = 15	*n* = 11	*n* = 9
Duration of first phloem phase ^B^ (h)	0.6 (±1.5)	2.2 (±3.0)	1.8 (±2.7)	1.4 (±2.5)	1.6 (±2.7)	3.7 * (±2.5)
Mean duration of sap ingestion ^B^ (h)	0.9 (±1.4)	2.8 (±2.8)	1.4 (±1.9)	1.3 (±2.0)	1.8 (±2.7)	3.7 * (±2.4)

^A^ Values are means ± SD; n = number of replications; *^,+^ significant difference in relation to control (**C**) and β-thujone (**2**), respectively (*p* < 0.05, Mann–Whitney U test); ^B^ only aphids that showed a phloem phase were used for analysis; ^a^ period when aphid stylets were withdrawn from plant tissues; ^b^ C+G+F; ^c^ E1+E2; ^d^ a probe includes all probing activities from the insertion of stylets into plant tissues until the withdrawal of stylets; ^e^ E2 > 10 min; ^f^ E1+E2/C+G+E+F; ^g^ E1/E1+E2.

**Table 3:** Exploratory cell punctures in non-phloem tissues after application of (+)-β-thujone (**2**) and its derivatives: bisulfite adduct (**3**), lactone (**4**), oxime (**5**), lactam (**6**) ^A^. The same plant was used to maintain the aphid stock culture.

EPG Parameters	Compounds					
	Control	2	3	4	5	6
	*n* = 15	*n* = 15	*n* = 15	*n* = 15	*n* = 15	*n* = 15
Proportion of probes with potential drops ^a^ (%)	80.1 (±11.5)	75.2 (±24.3)	70.5 (±21.7)	76.0 (±21.2)	62.2 * (±21.5)	49.4 *^,+^ (±19.2)
**Short Potential Drops (pd-S)**						
Total number of pd-S (#)	126.7 (±64.8)	115.5 (±88.7)	123.5 (±72.3)	130.4 (±62.7)	108.8 (±58.0)	75.6 * (±51.5)
Number of pd-S during a single penetration (#)	7.0 (±2.8)	11.0 (±11.1)	7.6 (±3.5)	8.8 (±9.5)	8.1 (±6.4)	6.5 *^,+^ (±7.5)
Mean duration of a single pd-S (s)	5.1 (±1.6)	4.2 * (±0.4)	4.0 * (±0.4)	4.1 * (±0.4)	4.9 (±2.3)	4.9 (±2.0)
Total duration of subphase II-1 of pd-S (s)	301.1 (±229.0)	204.5 (±167.4)	196.4 (±110.3)	233.5 (±113.7)	185.7 (±97.5)	129.4 * (±82.0)
Total duration of subphase II-2 of pd-S (s)	146.4 (±72.1)	122.8 (±97.9)	120.9 (±65.6)	128.6 (±59.1)	116.3 (±67.6)	81.9 * (±57.0)
Total duration of subphase II-3 of pd-S (s)	209.4 (±128.2)	160.4 (±131.4)	160.2 (±82.1)	171.0 (±88.2)	187.3 (±109.4)	136.6 (±96.8)
**Long Potential Drops (pd-L)**						
Total number of pd-L (#)	6.3 (±4.2)	7.3 (±6.1)	4.9 (±2.6)	8.7 (±5.8)	5.3 (±4.4)	4.3 (±3.6)
Number of pd-L during a single penetration (#)	0.3 (±0.2)	0.5 (±0.4)	0.3 (±0.2)	0.4 (±0.2)	0.4 (±0.5)	0.2 (±0.2)
Mean duration of a single pd-L (s)	6.4 (±2.2)	5.3 (±2.4)	6.0 (±2.1)	5.6 (±2.0)	5.6 (±1.8)	5.8 (±2.6)
Total duration of subphase II-1 of pd-L (s)	10.7 (±7.4)	12.8 (±11.9)	7.2 (±4.0)	14.5 (±9.8)	8.5 (±7.4)	7.0 (±6.0)
Total duration of subphase II-2 of pd-L (s)	7.1 (±4.9)	8.3 (±7.7)	5.3 (±2.6)	8.3 (±5.8)	5.8 (±5.2)	5.0 (±4.5)
Total duration of subphase II-3 of pd-L (s)	26.9 (±23.1)	26.0 (±24.3)	17.3 (±8.6)	28.8 (±18.3)	19.1 (±17.3)	14.0 * (±12.4)

^A^ Values are means ± SD; *n* = number of replications; *^,+^ significant difference in relation to control (**C**) and β-thujone (**2**), respectively (*p* < 0,05, Mann–Whitney U test); ^a^ pd-S + pd-L.
